# Prognostic value of liver stiffness in HIV/HCV-Coinfected patients with decompensated cirrhosis

**DOI:** 10.1186/s12879-018-3067-z

**Published:** 2018-04-11

**Authors:** Leire Pérez-Latorre, Matilde Sánchez-Conde, Pilar Miralles, Juan Carlos López, Francisco Parras, Francisco Tejerina, Teresa Aldámiz-Echevarría, Ana Carrero, Cristina Díez, Margarita Ramírez, Isabel Gutiérrez, José María Bellón, Rafael Bañares, Juan Berenguer

**Affiliations:** 1 Unidad de Enfermedades Infecciosas/VIH, Hospital General Universitario Gregorio Marañón, Doctor Esquerdo 46, 28007 Madrid, Spain; 2 Instituto de Investigación Sanitaria Gregorio Marañón (IiSGM), Madrid, Spain; 3 Servicio de Enfermedades Infecciosas, Hospital Ramón y Cajal, Madrid, Spain; 4 Servicio de Aparato Digestivo, Hospital General Universitario Gregorio Marañón, Madrid, Spain; 5 Centro de Investigación Biomédica en Red en el Área Temática de Enfermedades Hepáticas y Digestivas (CIBEREHD), Madrid, Spain; 6 Facultad de Medicina, Universidad Complutense de Madrid, Madrid, Spain

**Keywords:** Transient elastography, Liver stiffness, Liver fibrosis, FibroScan, Coinfection, HIV infection, Hepatitis C

## Abstract

**Background:**

Little is known about the utility of transient elastography (TE) for assessing the prognosis of patients with decompensated cirrhosis (DC).

**Methods:**

We analyzed HIV/HCV-coinfected patients with DC who underwent TE as part of their routine follow-up between 2006 and 2015. We also calculated the liver stiffness spleen diameter-to-platelet score (LSPS), FIB-4 index, albumin, MELD score, and Child-Pugh score. The primary outcome was death.

**Results:**

The study population comprised 65 patients. After a median follow-up of 32 months after the first TE, 17 patients had received anti-HCV therapy and 31 patients had died. The highest area under the receiver operating characteristic curve (AUROC) value for prediction of death was observed with albumin (0.695), followed by Child-Pugh score (0.648), both with *P* values < .05. Lower AUROC values were observed with MELD score (0.633), TE (0.618), LSPS score (0.595), and FIB-4 (0.569), all with *P* values > .05. In the univariate Cox regression analysis, albumin, FIB-4, Child-Pugh score, and MELD score, but not TE, were associated with death. In the multivariate analysis, albumin and Child-Pugh score were the only baseline variables associated with death.

**Conclusions:**

Our results suggest that TE is not useful for assessing the prognosis of HIV-infected patients with decompensated HCV-related cirrhosis. Albumin concentration and Child-Pugh scores were the most consistent predictors of death in this population group.

## Key points

Transient elastography was not useful for assessment of the prognosis of HIV-infected patients with decompensated HCV-related cirrhosis. Serum albumin concentration and Child-Pugh score were the most consistent predictors of death in this population group.

## Background

Chronic hepatitis C is more frequent and progresses more quickly in patients coinfected with human immunodeficiency virus (HIV) and hepatitis C virus (HCV) than in patients infected solely by HCV. In addition, progression to liver cirrhosis and decompensated liver disease is faster among the former [1].

Assessment of prognosis is an essential element of the care of patients with HCV-related cirrhosis, as it provides useful information for clinical management and treatment decisions. It is important to bear in mind that cirrhosis is not a final, static, and irreversible disorder but a dynamic and potentially reversible condition that comprises two major stages, compensated and decompensated disease, with marked differences in prognosis between them [2, 3]. Patients with compensated disease have a much longer median survival time than those with decompensated disease. Accordingly, measures of prognosis are also different for each stage; liver-related events such as hepatic decompensation or hepatocellular carcinoma are the most relevant outcomes in compensated cirrhosis, whereas death is the most relevant outcome in decompensated cirrhosis.

Portal pressure, which is usually determined using the hepatic venous pressure gradient (HVPG), is the most accurate predictor of liver-related events in patients with compensated cirrhosis [4]. In decompensated cirrhosis, survival is primarily related to liver function, which is usually assessed with scoring systems such as the Child-Pugh score and the Model for End-Stage Liver Disease (MELD) score [5].

The aim of our study was to assess the utility of liver stiffness as a prognostic indicator in HIV/HCV-coinfected patients with decompensated cirrhosis. We chose this objective for two reasons: first, because transient elastography has been found to be an accurate method for the non-invasive assessment of HVPG in patients with compensated liver disease [6, 7] and is potentially useful for prediction of clinical outcomes in these patients [8–10]; and second, because HVPG, in addition to the MELD score, has been independently associated with survival in patients with decompensated cirrhosis [11].

## Methods

### Study population and variables

We studied all consecutive HIV/HCV-coinfected patients with decompensated cirrhosis who underwent transient elastography as part of their routine follow-up between 2006 (when this technique was routinely introduced for staging of liver fibrosis in HIV/HCV-coinfected patients in our institution) and 2015. We extracted all the data from hospital records. Liver decompensation was defined based on a history of ascites, hepatic encephalopathy, variceal bleeding, or non-obstructive jaundice.

Transient elastography was performed by trained operators using a FibroScan® device (EchoSens, Paris, France). A median of 10 successful acquisitions was considered the representative measurement of liver stiffness. We considered 10 acquisitions with a success rate ≥ 60% and an interquartile range (IQR) < 30% of the median value as representative measurements.

We also calculated the liver stiffness spleen diameter-to-platelet score (LSPS) (liver stiffness × spleen diameter in cm/platelet count in 10^9^/l), as described elsewhere [12], and the FIB-4 index ([Age-years × AST-U/L]/ [Platelet count-10^9^/L × (ALT-U/L)^1/2^]), a non-invasive fibrosis index derived from patients included in the APRICOT trial [13] that has better prognostic value than liver biopsy for prediction of liver-related events in HIV/HCV-coinfected patients with compensated liver disease [14]. The length of the spleen was calculated by measuring the larger diameter observed in the imaging tests (ultrasound or computed tomography) performed closest to the transient elastography study during the previous year. The Ethics Committee of Hospital General Universitario Gregorio Marañón approved the study.

### Statistical analysis

Baseline was defined as the date of the first transient elastography determination after the diagnosis of decompensated cirrhosis. The length of the study was calculated for each patient from the date of the first transient elastography study to the last follow-up visit or death. The censoring date was June 30, 2015.

Categorical data were expressed as whole numbers and percentages and continuous variables as median and interquartile range (IQR). We used receiver operating characteristic (ROC) curves to determinate the ability of transient elastography to predict death.

Univariate Cox regression analysis was used to assess the association between transient elastography and other baseline covariates and death. As some patients were treated for HCV infection during follow-up, anti-HCV therapy was also included as a time-dependent variable in the univariate analysis. Multivariate Cox regression analysis was performed with the baseline variables that had attained a *P* value < .1 in the univariate analysis.

## Results

### Baseline characteristics

The study population comprised 65 HIV/HCV-coinfected patients with decompensated cirrhosis whose baseline characteristics, including the type of first hepatic decompensation, are shown in Table 1. The median date of first decompensation was August 2, 2008. The median time (IQR) from the first episode of decompensation to the first transient elastography was 6 (0–33) months.

**Table 1:** Baseline characteristics of 65 HIV/HCV-coinfected patients with decompensated cirrhosis categorized according to receipt of anti-HCV treatment during follow-up

Baseline characteristic	Patients who received HCV treatment (*n* = 17)	Patients who did not receive HCV treatment (*n* = 48)	All patients (*N* = 65)
Male sex - n (%)	13 (76)	30 (62)	43 (66)
Age, years – median (IQR)	46 (42–49)	46 (43–50)	46 (43–50)
HIV acquired by IDU – n (%)	12 (70)	45 (94)	57 (88)
Nadir, cells/mm^3^ – median (IQR)	199 (76–292)	121 (45–225)	136 (52–242)
CD4+ cells/mm^3^ – median (IQR)	294 (216–482)	258 (160–423)	293 (165–424)
CDC disease category C (%)	6 (35)	16 (33)	22 (34)
cART (%)	16 (94)	40 (83)	56 (86)
HIV-RNA < 5 0 copies/mL (%)	10 (59)	37 (77)	47 (71)
HBsAg-positive – n (%)	0 (0)	5(10)	5 (8)
Alcohol intake > 50 g/day – n (%)	2 (12)	11 (23)	13 (20)
First decompensation – n (%)			
Ascites	14 (82)	40 (83)	54 (83)
Variceal bleeding	2 (12)	3 (6)	5 (8)
Hepatic encephalopathy	0 (0)	5 (10)	5 (8)
Non-obstructive jaundice	1 (6)	0 (0)	1 (1)
Hepatocellular carcinoma – median (IQR)	1 (6)	2 (4)	3 (4.6)
Esophageal varices – n (%)			
Yes	5 (29)	25 (52)	30 (15)
No	8 (47)	9 (19)	17 (2)
Unknown	4 (23)	14 (29)	18 (28)
Child-Pugh score – median (IQR)	7 (5–9)	7 (6–8)	7 (5–8)
MELD score – median (IQR)	11 (7.5–15.5)	11 (7–14)	11 (7–14)
Serum albumin, g/dL – median (IQR)	3.5 (2.4–4.2)	3.0 (2.5–3.7)	3.1 (2.5–3.9)
FIB-4 value – median (IQR)	6.5 (3.1–13.5)	6.4 (4.2–10.6)	6.4 (3.8–10.6)
Liver stiffness, kPa – median (IQR)	50 (34–70)	48 (35–63)	48 (35–64)
LSPS – median (IQR)	8.8 (4.9–17.4)	10.5 (8–16.3)	10.3 (7.6–16.4)
HCV genotype – median (IQR)			
1	12 (70)	26 (54)	38 (58)
2	0 (0)	1 (2)	1 (1.5)
3	3 (18)	9 (19)	12 (18.5)
4	2 (12)	3 (6)	5 (7.7)
Unknown	0 (0)	9 (19)	9 (13.8)

*Abbreviations*: *HCV* hepatitis C virus, *IQR* interquartile range, *HIV* human immunodeficiency virus, *CDC* Centers for Disease Control and Prevention, *cART* combination antiretroviral therapy, *HBsAg* hepatitis B surface antigen, *LSPS* Liver stiffness x spleen diameter-to-platelet ratio

### Follow-up

The median follow-up was 32 months. A total of 31 (48%) patients died a median of 23 (13–51) months after the first transient elastography study; there were 19 liver-related deaths, 9 non-liver-related non-AIDS-related deaths, and 3 deaths of unknown cause. Four patients (6%) were diagnosed with incident hepatocellular carcinoma, and 34 (52%) patients had a new episode of liver decompensation that required hospitalization. No patients underwent liver transplantation.

During follow-up, 17 patients received anti-HCV therapy (simeprevir plus sofosbuvir [*n* = 4], pegylated interferon plus ribavirin [*n* = 4], pegylated interferon plus ribavirin and telaprevir [*n* = 3], daclatasvir plus sofosbuvir [*n* = 3], and ledipasvir plus sofosbuvir [*n* = 3]). Five patients achieved sustained viral response, 4 were non-responders, and 8 were on treatment or had not reached the time for assessment of treatment response at the censoring date.

### Predictors of mortality

The accuracy of liver stiffness and other baseline variables for the prediction of death are shown in Table 2. The highest area under the ROC curve (AUROC) value was observed with serum albumin concentration, followed by Child-Pugh score. Lower AUROC values were observed with MELD score, liver stiffness, LSPS score, and FIB-4 index.

**Table 2:** Accuracy of liver stiffness and other prognostic variables for the prediction of death assessed by ROC curves

Variable	Patients	AUROC (95% CI)	*P* Value
Serum albumin	64	0.695 (0.566–0.825)	.007
Child-Pugh score	65	0.648 (0.514–0.782)	.040
MELD score	65	0.633 (0.494–0.772)	.065
Liver stiffness	65	0.618 (0.482–0.755)	.100
LSPS	55	0.595 (0.443–0.747)	.225
FIB-4	64	0.569 (0.428–0.710)	.343

*Abbreviations*: *ROC* receiver operating characteristic, *AUROC* area under receiver operating characteristic curve, *CI* confidence interval, *MELD* model for end-stage liver disease, *LSPS* liver stiffness x spleen diameter-to-platelet ratio

The variables found to be associated with death by univariate Cox regression analysis were serum albumin, FIB-4 index, MELD score, and Child-Pugh score. Transient elastography almost reached statistical significance, with an unadjusted hazard ratio (HR) (95%CI) of 1.017 (0.998–1.037), *P*=. 078 (Table 3).

**Table 3:** Univariate Cox regression analysis: variables associated with death in 65 HIV/HCV-coinfected patients with decompensated cirrhosis

Variable	Univariate Analysis		
	HR	95% CI	*P*
Sex (male vs female)	0.887	0.417–1.887	.755
Age (per year)	1.011	0.943–1.085	.749
Year of decompensation (≤2008 vs > 2008)	1.238	0.563–2.724	.596
Alcohol intake > 50 g/day (yes vs no)	1.232	0.522–2.905	.634
HBsAg (positive vs negative)	1.210	0.366–4.005	.754
Nadir CD4+ (per cell/mm^3^)	1.000	0.997–1.003	.920
Baseline CD4+ (per cell/mm^3^)	0.999	0.997–1.001	.489
Esophageal varices (yes vs no)	0.729	0.336–1.582	.424
Liver stiffness (per kPa)	1.017	0.998–1.037	.078
Serum albumin (per g/dL)	0.482	0.298–0.780	.003
FIB-4 (per unit)	1.095	1.024–1.171	.008
Child-Pugh score (per unit)	1.356	1.100–1.671	.004
MELD score (per unit)	1.111	1.022–1.209	.014
LSPS (per unit)	1.023	0.987–1.060	.220
Anti-HCV therapy^a^ (no vs yes)	1.340	0.300–5.920	.695

*Abbreviations*: *HIV* human immunodeficiency virus, *HCV* hepatitis C virus, *HR* hazard ratio, *CI* confidence interval, *HBsAg* hepatitis B surface antigen, *MELD* model for end-stage liver disease, *LSPS* liver stiffness × spleen diameter/platelet ratio

^a^Computed as a time-dependent variable

Child-Pugh and MELD scores are composite indexes whose formulas both include bilirubin concentration. Therefore, considering that serum albumin is included in the calculation of the Child-Pugh score and that both liver stiffness and FIB-4 are non-invasive markers of liver fibrosis, we avoided collinearity by constructing four Cox multivariate models with variables that achieved a *P* value < .1 in the univariate analysis. As shown in Table 4**,** liver stiffness was not associated with death in any of the four models in which it was included. However, both serum albumin and Child-Pugh score were associated with death.

**Table 4:** Multivariate Cox regression analysis: variables associated with overall death in 65 HIV/HCV-coinfected patients with decompensated cirrhosis

	aHR	95% CI	*P*
Model 1			
Liver stiffness (per kPa)	1.013	0.991–1.034	.249
Serum albumin (per g/dL)	0.530	0.280–1.001	.050
FIB-4 (per unit)	1.027	0.932–1.131	.591
MELD (per unit)	0.986	0.872–1.115	.819
Model 2			
Liver stiffness (per kPa)	1.013	0.999–1.033	.221
FIB-4 (per unit)	1.028	0.936–1.129	.568
Child-Pugh score (per unit)	1.276	0.975–1.672	.076
Model 3			
Liver stiffness (per kPa)	1.013	0.992–1.035	.225
Serum albumin (per g/dL)	0.498	0.275–0.904	.022
MELD (per unit)	0.995	0.885–1.119	.936
Model 4			
Liver stiffness (per kPa)	1.014	0.995–1.034	.152
Child-Pugh score (per unit)	1.338	1.080–1.656	.008

*Abbreviations*: *HIV* human immunodeficiency virus, *HCV* hepatitis C virus, *aHR* adjusted hazard ratio, *CI* confidence interval, *MELD* model for end-stage liver disease, *kPa* kilopascal

## Discussion

In this study of 65 HIV/HCV-coinfected patients with decompensated cirrhosis, of whom approximately half had died after a median follow-up of 32 months, we did not find an association between liver stiffness and death. Moreover, the LSPS score, an index that has been found to outperform transient elastography in the prediction of portal hypertension and esophageal varices [12, 15, 16], was not superior to transient elastography for prediction of death. These findings are discordant with the marked ability of transient elastography to predict clinical outcomes such as liver-related events [8–10, 17] and death [8, 18] in patients with compensated HCV-related cirrhosis with or without HIV coinfection.

The baseline variables associated with death in the univariate analysis included serum albumin, FIB-4 index, Child-Pugh score, and MELD score. In the multivariate analysis, serum albumin and Child-Pugh score were the baseline variables that better predicted death in patients with decompensated cirrhosis; these findings are consistent with those of a systematic review of predictors of death in patients with cirrhosis [5]. Seventeen of the 65 patients in our study received anti-HCV therapy during follow-up; 10 received only oral direct-acting antivirals (DAAs). As some patients died before they could receive anti-HCV therapy, we analyzed anti-HCV as a time-dependent variable to avoid survivorship bias and found that it was not associated with survival. In a recent study, Foster et al. found that eradication of HCV with all oral DAAs was associated with short-term improvement in liver function in patients with HCV-related decompensated cirrhosis [19], although the long-term impact of eradicating HCV in this population group remains to be determined. Interestingly, the authors reported that serum albumin lower than 35 g/L was one of the baseline factors associated with lower likelihood of benefiting from therapy, in addition to old age and low serum sodium.

Our findings are best understood in the light of the major differences in the pathophysiology of compensated and decompensated cirrhosis. In compensated cirrhosis, portal pressure is the main determinant of prognosis, depends mainly on intrahepatic factors [20], and can be accurately estimated using transient elastography [6, 21]. However, prognosis in decompensated cirrhosis is determined to a large extent by extrahepatic factors, including activation of neurohormonal mechanisms and persistent bacterial translocation that activates the immune system and aggravates systemic inflammation [22]. These mechanisms contribute to the clinical manifestations characteristic of this stage of cirrhosis, including bacterial infections, hemodynamic derangement, and organ inflammatory damage, all of which contribute significantly to death [22].

The main limitation of our study is its retrospective design. However, it must be emphasized that patients were followed up by the same physicians throughout the course of the disease, with frequent clinical and laboratory monitoring, as is common practice with HIV-infected patients. In addition, the complications of cirrhosis were managed and prevented following protocols based on contemporary clinical practice guidelines. Another limitation of the study is the absence of information about alcohol and drug use during follow-up. This limitation is relevant, because alcohol and drugs are known modifiers of the natural history of HCV-related cirrhosis.

## Conclusions

In conclusion, our results suggest that transient elastography is not useful for assessing the prognosis of HIV-infected patients with decompensated HCV-related cirrhosis. Serum albumin concentration and Child-Pugh scores were the most consistent predictors of death in this population group.

## Abbreviations

AUROC: area under the ROC curve
DAAs: direct-acting antivirals
HCV: hepatitis C virus
HIV: human immunodeficiency virus
HVPG: hepatic venous pressure gradient
IQR: interquartile range
LSPS: liver stiffness spleen diameter-to-platelet score
MELD: model for end-stage liver disease
ROC: receiver operating characteristic
